# Laser Fabrication of Graphene-Based Electronic Skin

**DOI:** 10.3389/fchem.2019.00461

**Published:** 2019-06-27

**Authors:** Yu-Qing Liu, Zhao-Di Chen, Jiang-Wei Mao, Dong-Dong Han, Xiaoying Sun

**Affiliations:** ^1^State Key Laboratory of Integrated Optoelectronics, College of Electronic Science and Engineering, Jilin University, Changchun, China; ^2^College of Communication Engineering, Jilin University, Changchun, China

**Keywords:** laser, graphene, laser reduced graphene oxides, laser induced graphene, electronic skin

## Abstract

Graphene is promising for developing soft and flexible electronic skin. However, technologies for graphene processing is still at an early stage, which limits the applications of graphene in advanced electronics. Laser processing technologies permits mask-free and chemical-free patterning of graphene, revealing the potential for developing graphene-based electronics. In this minireview, we overviewed and summarized the recent progresses of laser enabled graphene-based electronic skins. Two typical strategies, laser reduction of graphene oxide (GO) and laser induced graphene (LIG) on polyimide (PI), have been introduced toward the fabrication of graphene electronic skins. The advancement of laser processing technology would push forward the rapid progress of graphene electronic skin.

## Introduction

As an ultrathin carbon material, graphene that features high conductivity, flexibility, transparency, bio-compatibility, and mechanical robustness have revealed great potential for developing soft and flexible electronics, especially, electronic skins (Badhulika et al., 2015; Han et al., 2016; Liao et al., 2018; Zeng et al., 2018; Zhu et al., 2018). To date, although graphene has been successfully prepared by mechanical exfoliation (e.g., tape method), chemical vapor deposition (CVD) on metal substrates (e.g., Cu and Ni), epitaxial growth, carbonization of polymers (e.g., laser induced graphene on polyimide, PI), solvents exfoliation and chemical oxidation of natural graphite, the application of graphene in soft electronics is still at an early stage (Chang and Wu, 2013; Han et al., 2015a, 2017; Cheng et al., 2017; Ye et al., 2018b). At present, the processing of graphene for electronic skin remains a challenging task. For instance, large-area and high-quality graphene have been obtained from CVD method. However, from the view point mass production and practical usage, the preparation of CVD graphene is energy-consumption, and the subsequent processing procedures usually involve complex substrate transfer and graphene patterning (Song et al., 2015). In this regard, advanced technologies enabling graphene processing is quite important for the progress of graphene-based soft electronics.

As a mask-free and chemical free method, laser processing of graphene has been successfully employed for both the preparation of graphene and the fabrication of graphene-based electronic skins (El-Kady and Kaner, 2014; Kymakis et al., 2014; Han et al., 2015b). Laser processing has been firstly used for the reduction and patterning of chemically derived graphene oxide (GO) (Zhang et al., 2014; Han et al., 2018; You et al., 2018). After laser treatments, the oxygen containing groups (OCGs) on GO sheets can be effectively removed, and therefore the conductivity can be recovered (Xiong et al., 2015; Hong et al., 2016). At the same time, any desired micropatterns can be directly created without the use of masks. Since the conductivity of laser reduced GO (LRGO) can be tuned by varying the laser intensity, laser reduction of GO has revealed great potential wearable soft sensors that can monitoring the human health (Zhao et al., 2017). Besides, laser processing has been extended to other graphene sources for patterning, structuring, layer control, heteroatom doping, and even device integration (Liu et al., 2016; Yoo et al., 2016). In recent years, laser treatment of PI has been proven an effective route for direct transform the polymer into grapheme (Ye et al., 2018a). Laser induced graphene (LIG) holds great promise for developing graphene-based electronic skins, since it is cost effective and permits flexible patterning.

**Figure 1:**
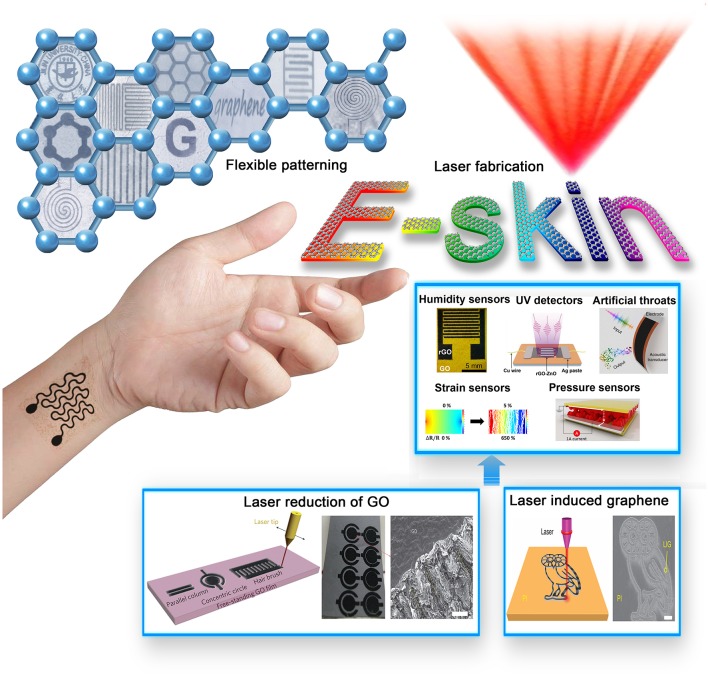
Laser fabrication of graphene-based electronic skin. Reproduced from Guo et al. (2014) with permission of WILEY-VCH. Reproduced from An et al. (2017) with permission of American Chemical Society. Reproduced from An et al. (2018) with permission of WILEY-VCH. Reproduced from Qiao et al. (2018) with permission of American Chemical Society. Reproduced from Tian et al. (2015) with permission of Nature Publishing Group. Reproduced from Tao et al. (2017) with permission of Nature Publishing Group. Reproduced from Gao et al. (2011) with permission of Nature Publishing Group. Reproduced from Lin et al. (2014) with permission of Nature Publishing Group.

In this minireview, we summarized the recent advancements in laser fabrication of graphene-based electronic skins (Figure 1). Unique features of laser reduction of GO and LIG technologies have been introduced, typical laser enabled graphene electronic skins have been reviewed. Current challenges and future perspective of this field have been briefly discussed.

## Laser Reduction of GO

Laser induced GO reduction can be attributed to either photochemical or photothermal effects. Smirnov et al. investigated the threshold which light (λ ≤ 390 nm) is able to trigger the deoxygenation of GO through the photochemical way (Smirnov et al., 2011). As a pioneer in this field, Zhang et al. demonstrated LRGO using 800 nm femtosecond laser (Zhang et al., 2010a,b). Most of the OCGs were removed at the laser scanned region. LRGO micro-circuits (line width, 500 nm) have been firstly created through a mask free manner. Subsequently, the conductivity and bandgap were tailored by controlling the content of residual OCGs, which can be realized by exposing to different laser power (Guo et al., 2012b). The resistivity of LRGO is significantly reduced to 3.91 × 10^−5^ Ωm from insulation. Besides, GO film can be reduced by other lasers, for instance, a CO_2_ laser (Gao et al., 2011). Owing to the thermal effects, the resultant LRGO is highly porous. Similarly, LightScribe DVD optical drive was used to reduce GO (El-Kady et al., 2012). The resulted LRGO nanosheets resistance can be tailored by the grayscale color and laser irradiation times (Strong et al., 2012). In addition to flexible patterning, laser treatment also enables hierarchical structuring, which leads to anisotropic and conductivity (Guo et al., 2012a; Wang et al., 2012; Jiang et al., 2014). On the basis of these results, laser processing technology has been employed for developing soft electronic skins (El-Kady and Kaner, 2013).

## Laser Induced Graphene

Recently, laser induced graphene has received great attention for their low cost, large-area, chemical-free and mask-free patterning. Tour's group found that porous graphene films were made from polyimide (PI) *via* CO_2_ laser irradiation (Lin et al., 2014). Upon laser irradiation, the C-O, C = O and N-C bonds of PI were broken due to the laser induced high temperature (>2,500°C), in this case, graphene formed through the recombination of atoms. The resultant LIG shows remarkably low sheet resistance (<15 Ω/sq) compared with PI. In addition to PI, other materials were also used to prepare graphene through a similar manner, such as wood (Ye et al., 2017), cloth (Chyan et al., 2018), polysulfone-class polymers (Singh et al., 2018). These methods provide a simple approach to fabricate graphene-based electronic skins.

## Graphene-Based Electronic Skin

Based on LRGO or LIG, various graphene-based electronic skins have been developed. Taking advantage of the laser direct writing technologies, the properties of graphene-based electronic skin can be tuned by laser power, scanning paths, and scanning speeds. The resulted porous and patterned graphene is helpful for design of graphene-based electronic skin. In this section, we will briefly summarize some typical applications of LRGO and LIG in electronic skins.

### Humidity Sensors

An et al. fabricated all-graphene based non-contact moisture-responsive electronic skin matrix (An et al., 2017). The highly reduced LRGO acts as the electrodes and the GO acts as the sensing materials. In this case, the humidity sensor enables direct measuring human breath and the distance between fingers and humidity sensors. In addition, a 4 × 4 sensing matrix was fabricated *via* laser direct writing technologies, which can detect the wet tip by the distribution of ΔZ/Z_0_. Moreover, the response and recovery time can be tailored by the OCGs contents of LRGO (Guo et al., 2012a).

### UV Detectors

An et al. produced LRGO-ZnO composites-based UV detectors *via* single-step and selective laser writing (An et al., 2018). The high writing speed was used to prepare the electrodes and medium writing speed was used to prepare the sensing materials. The porous LRGO-ZnO composites can accelerate the photoresponse of the device, in which the response and recovery time are about 17.9 and 46.6 s, respectively. This flexible UV light detector can act as wearable electronics and prevent human body to the overexposure of UV light.

### Strain Sensors

Wang et al. fabricated strain sensors based on the self-locked overlapping LRGO sheets (Wang et al., 2016). The strain sensor owns high gauge factor (>400). Furthermore, LRGO epidermal electronic skin was developed based on the crack directions and electrical characteristics (Qiao et al., 2018). The epidermal electronic skin (ultrahigh gauge factor of 673) is fabricated by the lift-off process based on based on laser scribed graphene. In this way, it can detect the respiration signal, pulse signal, and different finger bending degrees.

### Pressure Sensors

Tian et al. developed graphene-based resistive pressure sensor with a sensitivity of 0.96 kPa^−1^ in the range from 0 to 50 kPa (Tian et al., 2015). This pressure sensor is made of two LRGO film, which are perpendicular to each other. When force was applied on the LRGO film, the contact area increases, and the electrical path ways become more. Therefore, the current increases with a fixed voltage. It can detect pressing, bending, twisting forces.

### Temperature Sensors

Temperature response is another function of skin (Harada et al., 2014; He et al., 2015; Zhang et al., 2015; Cao and Wang, 2016; Trung et al., 2016). Sun et al. fabricated gas-permeable temperature sensors based on laser induced grapheme (Sun et al., 2018). This temperature sensor has ability in capturing temperature change of the skin, which the resistance changes as a function of temperature change (the error standard deviation, 0.25°C).

### Artificial Throats

Tao et al. reported an LIG-based intelligent artificial throat (Tao et al., 2017). LIG can detect throat vibration based on the piezoresistive property and work as a sound source based on the thermoacoustic property. Therefore, LIG-based intelligent artificial throat can clearly distinguish cough, hum and scream with different tones and volumes. Importantly, this artificial throat enables voice recognition. The sound pressure level has been demonstrated from 100 to 40 kHz.

## Conclusion and Outlook

Here, we have summarized two promising protocols for preparation and processing of graphene, LRGO and LIG. Taking advantage of the programmable processing manner of laser technologies, mask-free and chemical free patterning of graphene can be realized, revealing great potential for developing graphene-based electronic skins. The processing efficiency of laser fabrication is actually the key of large-scale applications. And the processing efficiency remains to be improved by spatial light modulator. Besides, the further trend of laser fabrication of graphene-based electronic skin can be integrated with various functional devices and/or reducing the device size. We believe that continued efforts in laser fabrication of graphene would push forward the rapid progress of this field and lead to breakthrough in both fundamental investigations and practical applications.

## Author Contributions

All authors listed have made a substantial, direct and intellectual contribution to the work, and approved it for publication.

### Conflict of Interest Statement

The authors declare that the research was conducted in the absence of any commercial or financial relationships that could be construed as a potential conflict of interest.
